# Specific Gene Loci of Clinical *Pseudomonas putida* Isolates

**DOI:** 10.1371/journal.pone.0147478

**Published:** 2016-01-28

**Authors:** Lázaro Molina, Zulema Udaondo, Estrella Duque, Matilde Fernández, Patricia Bernal, Amalia Roca, Jesús de la Torre, Juan Luis Ramos

**Affiliations:** 1 Environmental Protection Department, Estación Experimental del Zaidín, Consejo Superior de Investigaciones Científicas. C/ Profesor Albareda 1, Granada, Spain; 2 Abengoa Research, Campus de las Palmas Altas, Sevilla, Spain; 3 Imperial College London, South Kensington Campus, London, United Kingdom; 4 Bio-Iliberis R&D, C/ Capileira 7, 18210 Peligros, Granada, Spain; Universidad Nacional de la Plata, ARGENTINA

## Abstract

*Pseudomonas putida* are ubiquitous inhabitants of soils and clinical isolates of this species have been seldom described. Clinical isolates show significant variability in their ability to cause damage to hosts because some of them are able to modulate the host’s immune response. In the current study, comparisons between the genomes of different clinical and environmental strains of *P*. *putida* were done to identify genetic clusters shared by clinical isolates that are not present in environmental isolates. We show that in clinical strains specific genes are mostly present on transposons, and that this set of genes exhibit high identity with genes found in pathogens and opportunistic pathogens. The set of genes prevalent in *P*. *putida* clinical isolates, and absent in environmental isolates, are related with survival under oxidative stress conditions, resistance against biocides, amino acid metabolism and toxin/antitoxin (TA) systems. This set of functions have influence in colonization and survival within human tissues, since they avoid host immune response or enhance stress resistance. An in depth bioinformatic analysis was also carried out to identify genetic clusters that are exclusive to each of the clinical isolates and that correlate with phenotypical differences between them, a secretion system type III-like was found in one of these clinical strains, a determinant of pathogenicity in Gram-negative bacteria.

## Introduction

Hospital-acquired infections, also known as nosocomial infections, negatively impact patients, place hospital staff at risk and have been increasing in frequency in recent years. In the United States, the Centers for Disease Control and Prevention estimated that approximately 1.7 million of these hospital-acquired infections contribute to 99,000 deaths each year [1]. In European hospital surveys, Gram-negative infections are estimated to account for two-thirds of the 25,000 deaths linked to hospital-acquired infections each year [2]. One example of a bacterium that causes nosocomial infections is the opportunistic pathogen *Pseudomonas aeruginosa*. Infections caused by *P*. *aeruginosa* are associated with considerable morbidity, prolonged hospitalization and mortality. The mortality caused by this microorganism is around 36% (United States, 22.5%; France, 37.6%; Germany, 41.7%; Spain, 46.9%; and Italy, 46.3%) [3]. Nosocomial infections can cause severe pneumonia [4] and infections of the urinary tract [5], bloodstream [6] and other parts of the body, such as the skin [7]. Many infections are difficult to target with antibiotics because antibiotic resistance is spreading among to Gram-negative bacteria. This antibiotic resistance is acquired from resistant strains, which are present in the patient’s own flora that have emerged during antibiotic treatment, and is transferred via bacterial mobile genetic determinants of resistance (plasmids and transposons), resulting in the horizontal transfer of genomic traits [8, 9].

*Pseudomonas putida* is ubiquitous in edaphic and aquatic niches [10]. Occasionally, members of this species have been found to colonize human tissues in immuno-depressed hospital in-patients [9, 11, 12] bearing catheters or biliary drainage tubes [13]. Recently, the genomes of members of this species have been sequenced and analyzed [14, 15]—the results of these analyses have revealed that this species is particularly able to adapt to specific niches. Genomic analyses have also shown that, evolutionarily, horizontal gene transfer played a key role in this adaptation process because many of the niche-specific functions were found to be encoded on defined genomic islands [16–19].

*Pseudomonas putida* strains are often multidrug resistant and *P*. *putida* clinical isolates are considered to be an environmental reservoir of resistance determinants. Dissemination of these multidrug resistance elements to human pathogenic bacteria or to other opportunistic pathogens represents a potential threat [9, 20, 21]. The availability of the sequences of *P*. *putida* genomes from clinical and environmental strains provides the opportunity to determine the genetic clusters involved in the conversion of a typical environmental microorganism into one that is capable of colonizing human tissues. This approach has been previously used with *P*. *aeruginosa* to determine virulence traits as well as genes that may enhance fitness in the specific environmental niches occupied by opportunistic human pathogens [22–25]. The approach has also been used with the plant pathogen *P*. *syringae* [26] and with *P*. *putida* to study the specific niche adaptation of environmental strains [14, 15, 27].

Because hospital isolates of *P*. *putida* have been studied in terms of their resistance to antibiotics (i.e., [9, 21, 28– 31]), and more recently in terms of their potential to colonize and cause human tissue damage [32] we chose to focus on identifying genes present within *P*. *putida* clinical isolates that mediate nosocomial infections and survival in hospital settings. To this end, we choose an array of environmental strains that are able to survive in different environments and with different living strategies. The environmental strains that we chose were: the rhizospheric colonizer KT2440 strain [33]; the plant growth promoter BIRD-1 strain [34, 35]; the soil isolate and toluene degrader F1 strain [14]; the water isolate and high concentration solvent tolerant DOT-T1E strain [36, 37]; the water isolate GB-1 strain [14]; the nicotine degrader S16 strain [38]; and the endophytic colonizer W619 strain [14]. The clinical strains that we chose to study are *P*. *putida* isolates from the Hospital of Besancon in France, which our group has previously studied. These are: HB13667, a strain that was isolated from an in-patient presenting a general bacteremia, is unable to grow at 42°C, displays a pattern of antibiotic resistance similar to KT2440 and did not exhibit any pathogenic effect [32]; H8234, a strain that was also isolated from an in-patient presenting bacteremia and is also unable to grow at 42°C, but exhibited tetracycline and gentamycin resistance [12], and had a deleterious effect in tissue cultures *in vivo* on rat skin, but not in insect larvae [32]; HB4184, a strain that was isolated from fibrosis cystic sputum that is able to grow at 42°C, and caused damage only *ex vivo* in human tissue cultures [32]; and HB3267, a strain that exhibits a broad resistance spectra, which was isolated from an in-patient that passed away [9]. This strain caused damage in all the model systems studied [32]. All of the selected strains were initially identified as members of the *P*. *putida* species using 16S RNA sequences. Additional multilocus sequence analysis of *gyrB*, *rpoD*, *trpF*, *edd* and *recA* genes confirmed that these clinical isolates are members of the *P*. *putida* species [9,32].

## Materials and Methods

Clinical strains HB13667 (blood smear) and HB4184 (sputum culture) were isolated on Mueller-Hinton agar (Bio-Rad, Marnes-La-Coquette, France) in the hematology unit of the Hospital of Besancon (France). Antibiograms were performed using the Kirby-Bauer disk method on the same medium. Isolation and antibiograms were performed as indicated by the French Society for Microbiology (CA-SFM recommendations, November 2007)

Genomic DNA was purified from strains HB13667 and HB4184 using the Wizard Genomic DNA purification kit. Whole genome sequencing was performed using the 454 pyrosequencing strategy technology (Macrogen) and assembled using the GS de novo assembler 2.3 (Roche) into 150 and 141 contigs (25× fold coverage). These contigs were ordered by comparison using BLASTn and Mauve [39] with the sequences from other available *P*. *putida* genomes (accession no. NC_002947.3, CP000712.1, CP000926.1, CP000949.1 and CP002290.1). Characteristics of the environmental and clinical strains are summarized in Table 1.

**Table 1:** Strains used in this study.

Strain	Size (Mb)	G+C %	Coding sequences	Function assigned	RNAs	Environment	Source of isolation and main characteristics	References
**KT2440**	6.2	61.5%	5536	72,80%	43	Rhizosphere	Isolated from garden, ability to use 3-methylbenzoate	[40]
**BIRD-1**	5.7	61.7%	5209	71,60%	86	Rhizosphere	Isolated from a garden soil in a culture medium without iron and with insoluble inorganic phosphate as a source of phosphorous	[34]
**DOT-T1E**	6.3	61.4%	5705	70,60%	68	Waste water	Isolated from waste water treatment, degradation and tolerance toluene	[41]
**F1**	6.0	61.9%	5252	73,90%	95	Soil	Isolated from a polluted creek, aromatic degradation	[42]
**GB-1**	6.1	61.9%	5410	66,88%	96	Fres water	Isolated from fresh water, robust manganese (Mn^2+^) oxidizer	[43]
**W619**	5.8	61.4%	5182	72,46%	97	Plant tissue	Isolated from *Populus trichocarpa deltoides* cv. ‘Hoogvorst,’ endophyta	[44]
**S16**	6.0	62.3%	5410	70,02%	86	Rhizosphere?	Isolated from a field under continuous tobacco cropping, in medium with nicotine as C and N source	[45]
**HB13667**	6.3	62.4%	5817	70,00%	68	Clinical isolate	Isolated from immuno-depressed patient with general bacteremia, similar antibiogram profile to KT2440, unable to grow at 42°C. No pathogenic effect described.	[32]
**H8234**	6.9	61.9%	6305	67,58%	86	Clinical isolate	Isolated from blood of a immuno-depressed patient with general bacteremia, resistance to gentamicin and tetracycline, unable to grow at 42°C. Deleterious effects *ex vivo* and *in vivo* tissues.	[12]
**HB4184**	5.9	61.7%	5466	70,12%	69	Clinical isolate	Isolated from sputum of immuno-depressed patient with cystic fibrosis, resistance to streptomycin, able to grow at 42°C. Only deleterious effects in *ex vivo* tissues.	[32]
**HB3267**	5.9	62.7%	5322	72,60%	91	Clinical isolate	Isolated from blood of a deceased patient (unknown cause of death), antibiotic multi-resistant, able to grow at 42°C. Deleterious effects in all the tissues and insect models.	[9]

Genomic DNA was automatically annotated using a program pipeline based on Glimmer 3.0 for gene prediction [46]. BLAST and RPS-BLAST was used for functional assignment of open reading frames (ORFs) based on sequence similarity to sequences deposited in the NR, SwissProt, COG, Pfam, Smart, and PRK databases [47]. Automatic annotations were manually curated with Rapid Annotation using Subsystem Technology (RAST; http://rast.nmpdr.org) [48] and re-annotated and deposited in GenBank (HB13667—accession number: LKKS00000000 and HB4184 accession number: LKKT00000000). The resulting list of genes was realigned by bidirectional confrontation with the complete available genomes used in this study (one by one), in order to localize possible gaps in the sequence (HB13667 and HB4184). The ends of contigs were compared with Blastn and only small gaps (>2 Kb) were detected.

Genome sequence comparisons to determine protein homology, common genes and genetic islands were performed using RAST software (cutoff e-20). All the genome sequences were re-annotated using the same software (annotation scheme, ClassicRAST) and sequences were assigned a function-based similarity to a known sequence in the KEGG Database using an e-cutoff value <e^−10^. Genes present only in clinical strain genomes or found exclusively in one clinical strain were manually compared using BLASTp—one by one—against all the sequences available in the database. Those presenting less than 70% of sequence identity with any *P*. *putida* environmental strain (cutoff value <e^−30^) were selected. Functions for these genes were assigned manually based on sequence and domain similarity to sequences deposited in the NR, SwissProt, COG, Pfam, Smart and PRK databases [47].

Phylogenetic studies of complete genomes were performed using Composition Vector Tree (http://tlife.fudan.edu.cn/cvtree/). These phylogenetic studies were carried out as recommended by the user manual (http://tlife.fudan.edu.cn/cvtree/help/index.html) with a K-peptide length of 6, as recommended by the authors for prokaryote organisms [49].

For individual proteins, gene comparisons using Phylogeny based on the Multilocus Sequence Analysis (MLSA) of *gyrB*, *rpoD*, *trpF*, *edd* and *recA* genes was carried out using Phylogeny.fr (http://www.phylogeny.fr/). This multimodal platform performs multiple alignment sequences using the MUSCLE algorithm (full processing model), carries out alignment curation using the Gblocks program (to eliminate poorly aligned positions and divergent regions), and constructs phylogenetic trees using PhyML (model WAG, statistical test alr), which was gamma distributed with invariant sites (G+I parameters) and bootstrap values. Each branch was calculated 500 times for visualizations of the phylogenetic tree using TreeDyn software [50].

For phenotype characterization, individual colonies of *P*. *putida* strains from LB medium plates were streaked onto M8 pregrowth (M8PG) medium plates (0.1% [wt/vol] glucose, 0.1 g/liter NH_4_Cl, 1 mM MgSO_4_, 0.6 mg/liter Fe-citrate, and micronutrients), and grown overnight at 30°C. Growth in M8PG permitted the depletion nutrient reserves such that the subsequent growth assays with different carbon, nitrogen, and sulfur sources were dependent on the nutritional sources provided. The biomass of the overnight plates described above was recovered from the plate and washed twice with M8PG without glucose and NH_4_Cl eliminate possible nitrogen and glucose rests. Cultures were adjusted to an optical density at 660 nm (OD_660_) of 0.05. To test different carbon sources cells were grown in M8PG without glucose adding each carbon source at a final concentration of 5 mM. To test different nitrogen sources cells were grown in M8PG without NH_4_Cl adding each nitrogen source at a final concentration of 5 mM. T test different sulfur sources cells were grown in M8PG medium without MgSO_4_ adding each sulfur source at a final concentration of 5mM. The wells of the microplates were filled with 200 μl of the cellular suspension. Positive-control wells consisted of full minimal medium containing glucose and NH_4_Cl; negative-control wells contained this full medium without cell inoculum.

All data recordings were performed using a type FP-1100-C Bioscreen C MBR analyzer system (OY Growth Curves Ab Ltd., Raisio, Finland) at 30°C, with continuous agitation. The turbidity was measured using a wideband filter at 420 to 580 nm every 60 min over a 12-h period. Each strain was assayed at least three times for each of the compounds tested, and plates were visually examined following each assay in order to verify the results. To validate the screening results, cultivations were also performed in 100-ml conical flacks with 20 ml of culture medium. Turbidities of cultures under these conditions were usually twice that seen in the microplates, which validates the high-throughput approach. For stress experiments the strains were inoculated into microplate wells containing LB liquid medium, diluted 1/2, with the corresponding stressor concentration.

Strain HB4184 forms lumps and thick biofilms in the used culture conditions for this reason we discard this strain for phenotypical studies.

## Results

### *P*. *putida* clinical isolate species fall into two different clades

We were able to distinguish two different clades within the clinical strains and a third clade that consisted of exclusively environmental strains based in multilocus sequence typing (MLTS) analysis using *gyrB*, RNA polymerase sigma factor *rpoD*, N-(5'-phosphoribosyl) anthranilate isomerase *trpF*, 6-phosphogluconate dehydratase *edd* and the recombinase A *recA* gene/protein sequences (S1 Fig) as previously described [51]. To further gain information on the relationship between clinical and environmental isolates, we analyzed the complete genomes of the clinical strains and those of the environmental ones (S2 Fig). Further a comparison of the global sequence average identity of the different genome-encoded proteins using the RAST server was carrued out (Table 2).

**Table 2:** Average of the global identity of proteins encoded by the genomes of different *P*. *putida* strains.

**KT2440**	**100.00**												
**BIRD1**	**86.30**	**100.00**											
**F1**	**88.26**	**86.99**	**100.00**										
**DOT-T1E**	**83.10**	**82.70**	**86.04**	**100.00**									
**W619**	76.38	74.08	76.62	76.30	**100.00**								
**H13667**	73.51	71.69	74.04	74.25	69.23	**100.00**							
**HB3267**	78.49	77.23	78.72	78.11	73.67	**92.27**	**100.00**						
**S16**	78.53	76.24	77.43	78.17	72.52	**87.20**	**85.60**	**100.00**					
***P*. *monteilli***	79.27	77.29	79.34	**80.94**	73.66	**88.93**	**87.06**	**92.70**	**100.00**				
**GB1**	**80.27**	78.24	**80.08**	**80.01**	73.23	79.90	78.86	78.97	79.09	**100.00**			
**H8234**	71.75	70.98	72.30	73.09	68.01	72.08	70.16	71.85	72.00	**82.16**	**100.00**		
**HB4184**	77.92	76.65	78.03	78.42	72.00	79.15	77.85	77.58	78.08	**82.87**	**80.14**	**100.00**	
**NBRC14164**	78.90	78.78	78.83	79.79	72.68	78.08	78.04	77.48	77.93	**82.29**	**85.43**	**81.79**	**100.00**
	**KT2440**	**BIRD1**	**F1**	**DOT-T1E**	**W619**	**H13667**	**HB3267**	**S16**	***P*. *monteilli***	**GB1**	**H8234**	**HB4184**	**NBRC14164**

Numbers indicate the % of sequence similarity. **Bold numbers, identity sequence values >80**.

These analyses revealed that clinically-isolated strains HB13667, HB3267 and the environmental strain S16 could belong to the same clade, because they exhibit a protein identity >85% (Table 2). One possible explanation for close relationship of S16 with the two clinical isolates of this clade could be a common ancestor of edaphic origin for all of them. It should be noted that *P*. *putida* POXN01, other strain isolated from rice fields [52] as S16, has high relationship with strains clade I (S1 Fig). HB4184 and H8234 were found to be placed within a different clade, together with the fresh water isolate GB-1; protein identities of these strains were at least >80%. H8234 has a close relative in the water strain SJ3 [43, 53] (S1 Fig), this fact could indicate that GB-1, SJ3 and H8234 in clade II could have the same origin.

The third clade only contains environmental strains (the rhizobacteria KT2440 and BIRD-1, the “degraders” F1, ND6 and DOT-T1E). Protein identities within this group were >82% (Table 2). The endophytic strain W619 is not closely related to any of the other *P*. *putida* strains studied and exhibited protein identity global averages that were lower than 80% (Table 2) [54].

Considering the above results, we propose that *P*. *putida* clinical strains are organized into two distinct clades and that some environmental strains have significant identities at the protein level with these clinical isolates, while a third clade of strains only contains environmental isolates.

### Functional differences in *P*. *putida* clinical and environmental isolates

To determine the genetic clusters that are specific to the *P*. *putida* clinical strains, we compared functional categories and numbers of genes between clinical and environmental strains (Table 3). This analysis revealed that seven categories of genes are more abundant in clinical strains, namely:

**Table 3:** Functional categories in environmental and clinical *P*. *putida* isolates.

Functional categories	KT2440	BIRD-1	DOT-T1E	F1	GB-1	W619	S16	HB13667	H8234	HB4184	HB3267
**Cofactors, Vitamins, Prosthetic Groups, Pigments**	387 (9.6)	314 (8.4)	321 (7.9)	380 (10)	361 (10)	374 (9.9)	311 (8.2)	331 (9.5)	343 (8)	304 (7.9)	307 (7.9)
**Cell Wall and Capsule**	183 (4.5)	191 (5.1)	196 (4.9)	155 (4.1)	137 (3.8)	168 (4.5)	176 (4.6)	174 (5)	196 (4.6)	176 (4.6)	177 (4.6)
**Resistance to biocides**	**124 (3.1)**	**88 (2.4)**	**111 (2.8)**	**86 (2.3)**	**29 (0.8)**	**94 (2.5)**	**103 (2.7)**	**154 (4.41)**	**152 (3.6)**	**132 (3.4)**	**137 (3.5)**
*Heavy metal homeostasis and resistance	81 (2)	46 (1.2)	59 (1.5)	64 (1.6)	14 (0.4)	69 (1.8)	65 (1.7)	112 (3.2)	110 (2.6)	94 (2.5)	89 (2.3)
**Potassium metabolism**	49 (1.2)	38 (1)	31 (0.8)	21 (0.6)	2 (0.1)	21 (0.6)	31 (0.8)	34 (1)	34 (0.8)	28 (0.7)	32 (0.8)
**Miscellaneous**	85 (2.1)	56 (1.5)	65 (1.6)	90 (2.4)	91 (2.5)	85 (2.3)	52 (1.3)	55 (1.6)	75 (1.8)	55 (1.4)	49 (1.3)
**Phages, Prophages, Transposable elements, Plasmids**	3 (0.07)	5 (0.13)	11 (0.27)	1 (0.03)	0 (0)	2 (0.05)	16 (0.42)	41 (1.18)	40 (0,94)	35 (0.91)	31 (0.8)
**Membrane Transport**	202 (5)	158 (4.2)	225 (5.6)	145 (3.8)	145 (4.0)	167 (4.4)	162 (4.3)	196 (5.6)	236 (5.5)	209 (5.5)	183 (4.7)
*Protein secretion system, Type II	10 (0.25)	10 (0.27)	10 (0,25)	10 (0.26)	10 (0.28)	36 (0.95)	10 (026)	19 (0.54)	17 (0.4)	10 (0,26)	21 (0,54)
*Protein and nucleoprotein secretion system, Type IV	18 (0.45)	19 (0.51)	28 (0.69)	18 (0.47)	19 (0.52)	19 (0.51)	18 (0.48)	27 (0.78)	54 (1.23)	28 (0,73)	27 (0.70)
*Secretion system type I-Fimbriae	5 (0.12)	5 (0.13)	5 (0.12)	5 (0.13)	5 (0.13)	5 (0.13)	5 (0.13)	7 (0.20)	5 (0.12)	5 (0.13)	7 (0.18)
**Iron acquisition and metabolism**	107 (2.7)	66 (1.8)	60 (1.5)	115 (3.0)	82 (2.3)	110 (2.9)	54 (1.4)	57 (1.6)	70 (1.6)	61 (1.6)	56 (1.4)
**RNA Metabolism**	251 (6.2)	132 (3.5)	138 (3.4)	209 (5.5)	206 (5.7)	213 (5.7)	154 (4.1)	152 (4.4)	140 (3.3)	148 (3.9)	149 (3.9)
**Nucleosides and Nucleotides**	131 (3.3)	125 (3.3)	138 (3.4)	126 (3.4)	132 (3.6)	138 (3.7)	126 (3.3)	129 (3.7)	136 (3.2)	131 (3.4)	127 (3.3)
**Protein Metabolism**	258 (6.4)	273 (7.3)	267 (6.6)	251 (6.6)	245 (6.8)	246 (6.6)	266 (7.0)	242 (6.9)	264 (6.2)	249 (6.5)	264 (6.8)
**Cell Division and Cell Cycle**	33 (0.81)	37 (1.00)	35 (0.87)	32 (0.84)	34 (0.94)	33 (0.88)	33 (0.87)	36 (1.03)	28 (0.66)	36 (0.94)	36 (0.93)
**Motility and Chemotaxis**	77 (1.9)	118 (3.2)	130 (3.2)	82 (2.2)	80 (2.2)	78 (2.1)	117 (3.1)	120 (3.4)	120 (2.8)	122 (3.2)	117 (3.0)
**Regulation and Cell signaling**	100 (2.5)	113 (3.0)	109 (2.7)	111 (2.9)	112 (3.1)	109 (2.9)	116 (3.1)	118 (3.4)	122 (2.9)	111 (2.9)	110 (2.8)
**Secondary Metabolism**	5 (0.12)	5 (0.13)	7 (0.17)	14 (0.37)	5 (0.13)	14 (0.37)	5 (0.13)	5 (0.14)	5 (0.12)	5 (0.13)	5 (0.13)
**DNA Metabolism**	139 (3.4)	125 (3.3)	140 (3.5)	133 (3.5)	127 (3.5)	130 (3.5)	140 (3.7)	154 (4.4)	140 (3.3)	151 (3.9)	143 (3.8)
**Fatty Acids, Lipids, and Isoprenoids**	136 (3.4)	178 (4.8)	188 (4.7)	147 (3.9)	128 (3.5)	140 (3.7)	169 (4.5)	195 (5–6)	200 (4.7)	185 (4.8)	185 (4.8)
* Phospholipids	35 (0.87)	44 (1.12)	45 (1.12)	37 (0.97)	38 (1.08)	34 (0.95)	47 (1.24)	48 (1.38)	54 (1.28)	47 (1.24)	48 (1.24)
**Nitrogen Metabolism**	16 (0.40)	35 (0.94)	39 (0.97)	34 (0.89)	23 (0.64)	25 (0.67)	33 (0.87)	31 (0.89)	36 (0.84)	33 (0.86)	33 (0.85)
**Dormancy and Sporulation**	5 (0.12)	3 (0.08)	4 (0.10)	5 (0.13)	4 (0.11)	5 (0.13)	3 (0.08)	3 (0.09)	4 (0.09)	3 (0.08)	3 (0.08)
**Respiration**	183 (4.5)	145 (3.9)	151 (3.8)	167 (4.4)	175 (4.8)	171 (4.6)	164 (4.3)	154 (4.4)	159 (3.7)	138 (3.6)	161 (4.1)
**Stress Response**	**190 (4.7)**	**180 (4.8)**	**186 (4.6)**	**170 (4.7)**	**170 (4.7)**	**181 (4.8)**	**184 (4.9)**	**198 (5.7)**	**204 (5.0)**	**195 (5.1)**	**194 (5.0)**
*Oxidative stress	95 (2.4)	85 (2.3)	87 (2.2)	64 (1.7)	58 (1.6)	76 (2.0)	80 (2.1)	95 (2.7)	91 (2.1)	88 (2.3)	89 (2.3)
*Osmotic stress	22 (0,54)	36 (0.96)	37 (0.92)	22 (0.58)	33 (0,91)	26 (0.69)	32 (0.84)	37 (1.06)	46 (1.08)	40 (1.04)	40 (1.04)
**Choline and Betaine Uptake and Betaine Biosynthesis	18 (0.47)	26 (0.70)	30 (0,74)	17 (0,45)	27 (0.74)	20 (0,53)	25 (0.66)	30 (0.86)	38 (0.89)	33 (0.86)	33 (0.85)
**Metabolism of Aromatic Compounds**	133 (3.3)	136 (3.6)	187 (4.6)	168 (4.4)	152 (4.2)	153 (4.1)	100 (2.6)	123 (3.5)	152 (3.6)	119 (3.1)	118 (3.1)
**Amino Acids and Derivatives**	614 (15.2)	616 (16.5)	646 (16.0)	563 (14.8)	555 (15.2)	571 (15.2)	629 (16.6)	636 (18.2)	689 (16.1)	635 (16.7)	647 (16.7)
*Cysteine Biosynthesis	18 (0.45)	26 (0.70)	26 (0.65)	20 (0.53)	21 (0.58)	20 (0.53)	26 (0.69)	28 (0.80)	27 (0.63)	28 (0.73)	27 (0.7)
*Glutamine, Glutamate, Aspartate and Asparagine Biosynthesis	27 (0.67)	35 (0.93)	41 (1.01)	28 (0.73)	34 (0.94)	29 (0.77)	41 (1.08)	42 (1.20)	43 (1.01)	41 (1.07)	44 (1.13)
*Histidine Degradation	7 (0.17)	8 (0,21)	8 (0.20)	8 (0.21)	8 (0.22)	8 (0.21)	8 (0.21)	9 (0.26)	8 (0.19)	9 (0.23)	9 (0.23)
**Sulfur Metabolism**	79 (2.0)	67 (1.8)	83 (2.1)	81 (2.1)	122 (3.4)	69 (1.8)	82 (2.2)	91 (2.6)	127 (3.0)	89 (2.3)	93 (2.4)
*Organic sulfur assimilation	45 (1.1)	29 (0.78)	44 (1.09)	43 (1.13)	83 (2.3)	30 (0.80)	43 (1.13)	51 (1.46)	86 (2.02)	54 (1.41)	54 (1–40)
**Phosphorus Metabolism**	59 (1.5)	48 (1.29)	49 (1.22)	55 (1.44)	59 (1.63)	48 (1.28)	49 (1.29)	55 (1.58)	52 (1.22)	50 (1.30)	49 (1.27)
**Carbohydrates**	457 (11.3)	445 (11.9)	471 (11.7)	443 (11.6)	417 (11.5)	387 (10.3)	464 (12.2)	444 (12.7)	509 (11.9)	438 (11.4)	440 (11.4)
**Total genes with functional category**	4029	3732	4028	3808	3618	3755	3788	3489	4261	3833	3864

Numbers indicate the number of genes involved in a given functional category. In parentheses, percentages considering the total of genes with functional category.

**Fig 1:**
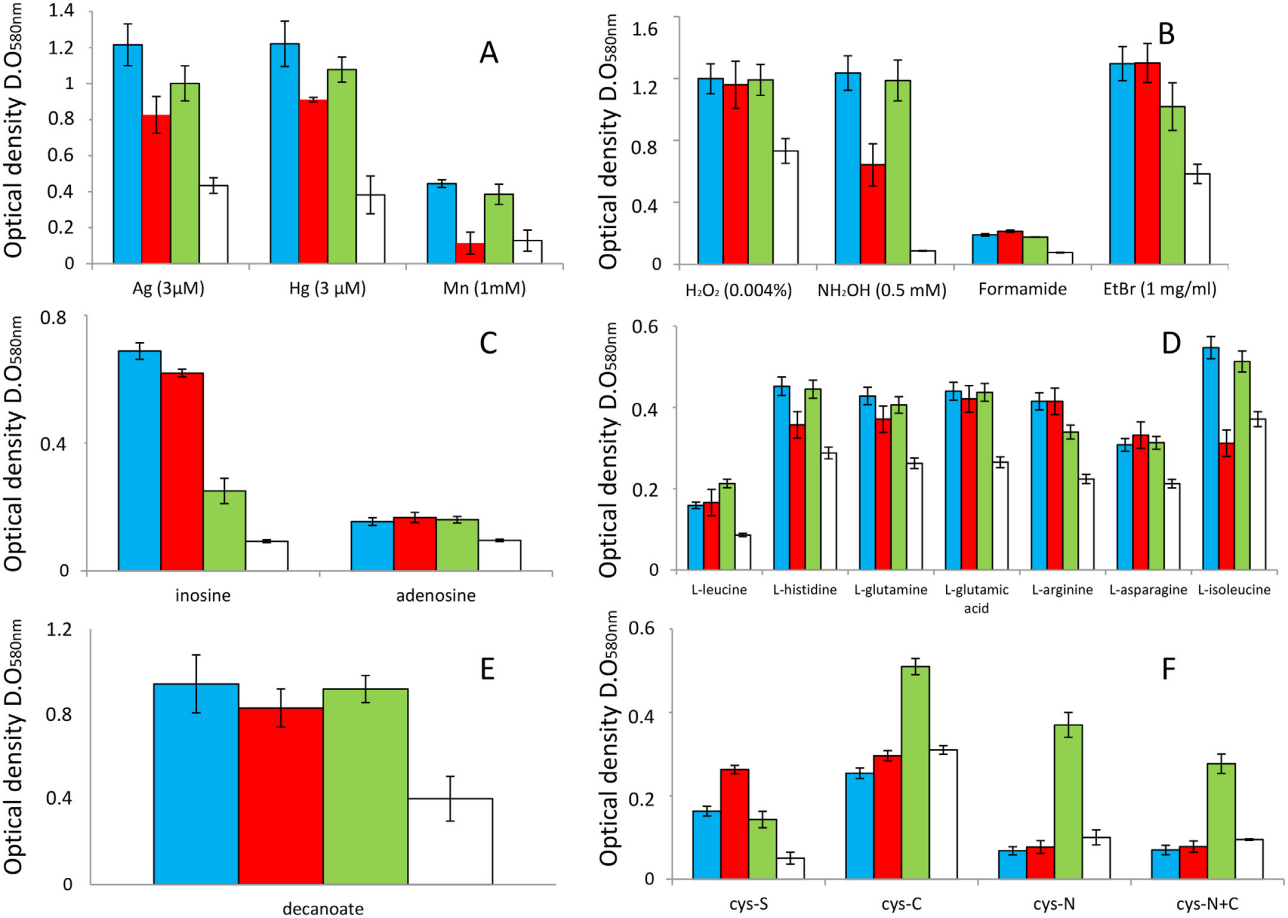
Phenotypical array characterization of clinical strains. Graphics show the growth of the studied *P*. *putida* clinical strains and KT2440 in the presence of heavy metals **(A)**; oxidative and other stressors **(B)**; DNA intermediates as the only nitrogen source **(C)**; amino acids **(D)** or fatty acid **(E)** as the only carbon source; and cysteine (cys) as the only sulfur (S), nitrogen (N), carbon (C) or carbon+nitrogen source (C+N). Blue bars, HB13667; red bars, H8234; green bars, HB3267 and white bars, KT2440. Error bars indicate standard deviation from three experimental repetitions. In parentheses concentration of stressor used, if concentration is not indicated means this was 5 mM. HB4184 was not included in this study because it forms lumps and thick biofilms in these culture conditions.

1. Genes that encode for antibiotic, antimicrobial and heavy metal resistance. These numbers varied in clinical strains from 154 in HB13667 to 132 in HB4184 (Table 3). For environmental strains these numbers were always below 124. The degree of resistance to a range of antibiotics by KT2440 was compared with the resistance to the same antibiotics of the set of clinical strains used in this study; in general, and in agreement with Fernandez et al., [32], the clinical strains were more resistant than KT2440. Phenotypic studies performed in this study demonstrated than clinical strains were also more resistant to some heavy metals as silver (3 μM) and mercury (0.3 μM) than KT2440; in both cases, the optical density reached by cultures of clinical strains was always at least 2-fold higher than the case of KT2440 (Fig 1).
2. Genes involved in DNA insertion events that integrate phage, prophage and transposable elements. In clinical strains these numbers are always over 31 and in environmental strains the maximal number found was 16 for the S16 strain (Table 3). One remarkable finding is that the percentage of inserted DNA in strain H8234, constitutes approximately 20% of the total DNA. We have shown in biparental mating between HB3267and KT2440 active transfer of genes codifying gentamycin and streptomycin resistance to KT2440 [9]. The frequency was on the order of 2x10^−8^ transconjugants per recipient cell what reveals that horizontal gene transfer events are clearly linked to the spread of antibiotics markers in the species of *P*. *putida*.
3. Genes associated with DNA metabolism, especially in HB13667 and HB4184 (Table 3). These genes are found in clinical strains in numbers over 140, what is above the number of these genes in environmental strains. Phenotypic studies demonstrated that clinical isolates grew in the presence of purines as nitrogen source (adenine and inosine), while the environmental strain KT2440 hardly could use these chemicals as N source (Fig 1).
4. Genes associated with the fatty acid metabolism. Here we have found that clinical strains, with the exception of HB4184, appear to have a higher number of genes involved in phospholipid metabolism than environmental strains (Table 3). Phenotypic analyses demonstrated than clinical strains are able to growth better than the environmental strain KT2440 using short chain lipids as decanoic acid as carbon source; the optical densities reached by cultures of clinical strains were at least 2-fold higher than KT2440 (Fig 1).
5. Genes involved in survival under stress conditions (Table 3). Genes that appear to be predominant in clinical strains compared with environmental strains are associated with handling oxidative stress and osmotic stress; these include genes involved in biosynthesis and transport of choline—betaine. (Table 3). We have tested the resistance of the clinical strains and the environmental KT2440 to different oxidative agents as oxygen peroxide and hydroxylamine. Clinical strains grew in presence of hydroxylamine (0.5 mM), while KT2440 did not survive at this concentration (Fig 1). Clinical isolates survived in a proportion of 100% after exposition to 0.004% v/v of H_2_O_2_ (optical densities of cultures with and without out this compound were the same, 1.2), while the survival of KT2440 was reduced until a 70% by the presence of this compound (Fig 1). Clinical strains were able to resist better the presence of formamide (they are able to use this molecule as the sole N source) and ethidium bromide than the environmental KT2440 (Fig 1).
6. Genes involved in amino acid metabolism were more frequent in clinical strains than in the environmental ones, expect for DOT-T1E that had similar numbers in this category. Genes involved in cysteine and histidine degradation seem to be more abundant in clinical isolates than in environmental strains what may represent an adaptation to the human body (Table 3). Phenotypic studies demonstrated that clinical strains are able to use more efficiently the amino acids and derivatives leucine, histidine, glutamine, glutamic acid, asparagine, serine, amino valeric acid, amino butyric acid, glutaric acid and phenylethylamine as carbon source more efficiently than KT2440 (Fig 1 and not shown).
7. Genes involved in metabolism of organic sulfur (Table 3); ranged between 51 in HB13667 and 86 in H8234, while environmental strain have less than 45 with the only exception being GB-1, where 83 genes belonging to this category were identified (Table 3). In this regard, clinical strains were able to use L-cysteine as sulfur source, while KT2440 was not able to growth in these conditions (Fig 1).

Other phenotypical differences found in clinical strains are the capacity to grow better than KT2440 in short carboxylic acids (acetic acid, L-lactic acid, D, and L- malic acid, malonic acid, (methyl)-pyruvate, and succinic acid); cyclic compounds and derivatives with a high antioxidant power (quinic acid, benzoate, 4-hydroxybenzoate, protocatechuate); threalose, involved in desiccation and osmotic stress, where KT2440 was not able to grow; and the detergent Tween20 as carbon source (data not shown).

### Analysis of *P*. *putida* genes from clinical isolates

To more precisely discriminate the clinically important genes of *P*. *putida*, we analyzed the amino acid sequence identities of the ORFs found within the genomes of the clinical strains. Specifically we looked for ORFs which were present in clinical strains but not in environmental strains or ORFs that had an identity <70% with the environmental strains of *P*. *putida*. Genes shared by the two clades of clinical isolates are defined here “core clinical genes”.

#### *P*. *putida* “core” clinical genes, not present in environmental isolates

The most relevant characteristic of *P*. *putida* core clinical genes is that they are mostly present on transposons (Table 4); indicating their horizontal acquisition from other microorganisms. Many of these genes encode proteins which have a high degree of identity with proteins identified in the insect pathogen *Pseudomonas entomophila*, [55], and in the opportunistic pathogen *Pseudomonas aeruginosa* [56] (Table 4). The functions of the proteins encoded by genes in the transposons are related to survival under oxidative stress conditions, resistance against biocides, amino acid metabolism (specifically, histidine degradation) (Fig 1), and virulence (two toxin/antitoxin (TA) systems) (Table 3).

**Table 4:** Core and clade specific genes in *P*. *putida* clinical isolates.

Coordinates		General function	Assigned function	Clade I	Clade 2	Closest relative found in
**Clinical core genes**						
Genes involved in anti-immune response						
L483_14650-L483_14665	Transposon	Oxidative stress	Formaldehyde degradation, nitric oxide (NO) homeostasis	HB13667	H8234	*P*. *aeruginosa*
B479_11910-B479_11935	Transposon	Amino acid metabolism	Histidine degradation	HB3267, HB13667	HB4184	*P*. *entomophila*
Resistance to biocides						
L483_14890-L483_14955	Transposon	Virulence	Mercury resistance	HB13667	H8234	*P*. *aeruginosa*
L483_15590-L483_31960	Transposon	Virulence	Nickel, cobalt-zinc-cadmium and chromate resistance	HB13667	H8234, HB4184	*P*. *aeruginosa*
Iron uptake						
L483_16595- L483_16480	Transposon	Iron uptake	Alternative siderophore pathway	HB13667	H8234	*P*. *alcaligenes*
Bacteriocine production						
L483_14885 -L483_14910	Transposon	Virulence	Toxin (RelE)/antitoxin gene system	HB13667	H8234, HB4184	*P*. *aeruginosa*
**Clade I clinical genes**						
Genes involved in anti-immune response						
B479_12025		Transport	Manganese transport, cell invasion	HB3267, HB13667		*P*. *plecoglossicida*
B479_16565		Signalling	guanosine pentaphosphate, cell invasion	HB3267, HB13667		*Pseudomonas* sp. URMO17WK12:I9
Host/microbe interaction structures						
B479_21275-B479_21345		Virulence	Type II/IV secretion system (T4SS)	HB3267, HB13667		*P*.*plecoglossicida*
B479_12550-B479_12545		Virulence	TolC family type I secretion	HB3267, HB13667		*P*. *aeruginosa*
Bactericide production						
B479_00570-B479_00575		Virulence	Toxin/antitoxin system RelE	HB3267, HB13667		*P*. *syringae*
B479_01965-B479_01960		Virulence	S-type pyocin-based system	HB3267, HB13667		*P*. *plecoglossicida*
B479_22065-B479_22070		Virulence	S-type pyocin-based system	HB3267, HB13667		*Enterobacteriaceae*
B479_10725- B479_10755		Virulence	Microcystins are potent toxin	HB3267, HB13667		*Microcystis aeruginosa*
Tissue colonization						
B479_07330- B479_07355		Virulence	Type 1 pili tissue adherence, colonization and invasion	HB3267, HB13667		*P*. *putida* NBRC 14164T
B479_11885- B479_11910		C and aminoacid metabolism	Alternative sugars and the use of amino acids	HB3267, HB13667		*P*. *plecoglossicida*
B479_19460- B479_19480		Iron uptake	Siderophore biosynthesis	HB3267, HB13667		*P*. *plecoglossicida*
**Clade II clinical genes**						
L483_11530–90		Lipid metabolism	Alternative phospholipid biosynthesis pathway		H8234, HB4184	*P*. *entomophila*
L483_30550–60		Virulence	Type IV pili		H8234, HB4184	*P*. *monteilii*

Of note, Toxin-Antitoxin (TA) systems are not more numerous in the genome of clinical strains compared with environmental strains; in fact, the strain with the most TA systems is KT2440, the rhizospheric strain, with 9, while the rest of the strains possess 5 or 6 (S1 Table). Some of the TA systems are shared by the most of the strains e.g., toxin with a RES domain/antitoxin encoded in KT2440 by the genes PP_2433, PP_2434, was present in all the strains with the exception of W619. Others are niche specific; i.e. “clinical TA systems” e.g., antitoxin MazE family/hypothetical toxin encoded in HB3267 by the genes B479_25735 and B479_25740, present in all the clinical strains with the exception of HB13667 or the RelE/RelB TA system encoded by the genes L483_14885 -L483_14910 in H8234, present in all the clinical strains with the exception of HB3267. Overall the so-called “clinical” TA systems were present in most of the clinical strains but not at all in the environmental strains. “Environmental” TA systems e.g., the RNA interferase/antitoxin MqsA encoded in KT2440 by PP_4204- PP_4204, was present in most of the environmental strains with the exception of S16; or the TA system hicA-1/ hicB-1 encoded by PP_1479- PP_1480 in KT2440, was present in most of the environmental strains with the exception of GB-1. Therefore, some of the TA systems are specific at the level of the strain: 3 in KT2440, 2 in W619, 1 in DOT-1E, 2 in GB-1. A similar phenomenon is observed with other bacteriocins such as S-type pyocines (S1 Table), siderophore biosynthetic genes, and O-antigen production (data not shown).

#### Clade I clinical genes (HB13667 and HB3267)

Most of the genetic clusters shared by HB13667 and HB3267 strains, have homologs in pathogenic bacteria, in addition to the previously mentioned *P*. *entomophila* and *P*. *aeruginosa*, these include, the plant pathogens *Pseudomonas syringae*, also described as an opportunistic human pathogen [57], and *Pseudomonas amygdali* [58]; Enterobacteriaceae family; the harmful cyanobacteria *Microcystis aeruginosa* [59]; the fish pathogen *Pseudomonas plecoglossicida* [60]; and the clinical isolates *P*. *putida* NBRC 14164T [61] and *Pseudomonas monteilii* [62] (Table 4). These clusters encode proteins involved in transport of ions (Mn^++^) or peptides across the membrane (type I, II and IV secretion systems). These secretion systems, particularly type II and IV are more abundant in clinical strains than in environmental strains (Table 3). Three genetic clusters encoding bacteriocins (RelB/RelE TA system and two pyocin/immunity systems) and the genes involved in the siderophore pyoverdine of these strains seem to be specific for this clade (Table 4).

Phenotypic studies corroborated that clade I strains were able grow in presence of manganese (1μM), meanwhile clinical strains clade II and KT2440 were unable to growth at these conditions (Fig 1). Clade I clinical strains were able to use the amino acids histidine and isoleucine (Fig 1), and alternative carbon source as glycerol, D-fructose, propionic acid with more efficiency than clade II clinical strains and KT2440 (data not shown).

#### Genes that are specific to HB13667

At the level of functional categories, this strain stands out attending the number of genes involved in resistance to antibiotics and toxic compounds (Table 3, S2 Table), genes involved in DNA acquisition (Table 3, S2 Table), genes that encode proteins related with resistance of oxidative stress (sharing the first position with KT2440), DNA metabolism, cysteine biosynthesis (together with H8234) and polyhydroxybutyrate metabolism (S2 Table). This suggests the acquisition by HB13667 of genetic traits involved in the coping of selective pressure conditions (the presence of biocides and under oxidative stress conditions) from well adapted microorganisms that have shared the same habitat with this clinical isolate. Phenotypic assays corroborated that this strain was more resistant than the other clinical strains and KT2440 to oxidant molecules as dichromate, and heavy metals as silver and telluric acid. (Fig 1 and data not shown). When cells were exposed to this last compound (0,9 ug/ml) optical densities reached by HB13667 cultures were 2-fold higher than the rest of the studied strains.

Strain HB13667 possesses 21 genetic clusters (named I to XXI in S3 Table) that are specific for this strain (i.e., with identities lower than 70% compared to other strains). Within these clusters are a remarkable array of equipment for the detoxification of oxidative reactive species (e.g, genetic cluster I contains an alkylhydroperoxidase—ORF175, a catalase—ORF 244 and a peroxidase—ORF247) and to maintain beneficial redox conditions for biomolecules via glutathione metabolism; in genetic clusters I, II, X and XIV (S3 Table). Regarding the resistance of this strain to the presence of biocides, ORF4897-4903 found in cluster XVII encode for proteins involved in detoxification of heavy metals. Specifically, there is a transport system for cobalt/zinc/cadmium that is found within a transposon that has high identity to sequences in *P*. *aeruginosa* (S3 Table).

#### Genes that are specific to HB3267

This strain is the richest in genes involved in amino acid metabolism (glutamine, glutamate, aspartate, asparagine, threonine, homoserine, histidine and putrescine) compared with all the studied strains (S2 Table). One interesting aspect of this strain is that it possesses a high number of genes which encode for type II protein secretion systems, only over-passed by the endophytic W619 (Table 3, S2 Table); Another significant observation is that this strain has a high number of multidrug resistance/efflux systems (S2 Table). However, the most clear feature indicate that the major differential evolution strategy used by this strain is related to the metabolism of certain amino acids. Phenotypic characterization of this strain revealed that it was able to use L-cysteine as carbon, nitrogen or carbon+nitrogen source better than the rest of the studied clinical strains and KT2440 (Fig 1).

The exclusive genetic information found in this strain is grouped into twenty one genetic clusters on its chromosome (S3 Table). The most remarkable clusters are found within two transposons (S3 Table). The first of which is, cluster I, containing a *Tn7*-like transposon (B479_00025-B479_00140), with genes that share high identity with *P*. *aeruginosa* and *P*. *plecoglossicida*, for example B479_00040-B479_00065, encoding genes involved in D-amino acid metabolism. This transposon also bears genes (B479_00070- B479_00095) involved in the transport of glycine-betaine-proline which mediate survival under osmotic stress. In addition, there is B479_00100- B479_00105, which is involved in the transport of γ-aminobutyric acid (GABA). The second transposon (B479_25745-B479_25935, in genetic cluster XX) contains genes mainly involved in tolerance and degradation of toxic aromatic compounds (S3 Table) (9).

Other genes of interest, not located within transposons, include a set of genes (B479_12970-B479_13085, in genetic cluster XII) that share high identity with genes of *P*. *fluorescens* and *P*. *syringae* that encoded proteins that are involved in the transport and degradation/biosynthesis of cyclic/aromatic halogenated compounds. It should also be mentioned three potentially secretable proteins within this cluster namely: B479_13035 (a nitrilase), and B479_13040 and B479_13080 (two phospholipases) (S3 Table).

The phenotypic characterization of HB3267 revealed that this strain is thermo-resistant. Although this phenotype could be due to multiple elements, it should be noted that genetic cluster X contains a group of hypothetical proteins, one of which is a homolog to HSR1 (B479_10540) a heat shock protein.

#### Clade II clinical genes (H8234 and HB4184)

Specific genetic clusters shared by H8234 and HB4184, and absent in other *P*. *putida* strains, encode proteins that are homologous to those found in bacteria that are pathogens, such as, *P*. *entomophila* and microorganisms typically isolated from hospital environments for example members of the Enterobacteriaceae family and *P*. *monteilii* (Table 4). These genes are involved in lipid metabolism and membrane transport (Type IV secretion systems) (Table 4), genes which are also well represented clinical strains (Table 3).

In addition, it is important to highlight the existence of two phospholipids biosynthesis pathways in H8234 and HB4184; one of these (L483_11530–90) has a homolog in the entomopathogen *P*. *entomophila*, while the other (L483_17350- L483_17400) is highly conserved in *P*. *putida* strains. The presence of two phospholipid biosynthetic pathways is very common in human pathogenic strains.

#### Genes that are specific to H8234

H8234 was superior to the other strains in genes of multiple categories such as: cell wall and capsule biosynthesis (capsular and extracellular polysaccharides, LOS core and oligosaccharide biosynthesis), virulence, disease and defense, protein and nucleoprotein secretion system Type IV, regulation and cell signaling, metabolism of lipids (phospholipids, fatty acids and isoprenoids), stress response (osmotic stress), amino acids and derivatives metabolism (polyamine, arginine, ornithine, isoleucine, alanine, serine, and glycine), sulfur metabolism (organic sulfur assimilation) and carbohydrate metabolism (S2 Table). This suggests that the evolutionary strategy of this strain was much more complex than for the rest of the others. Comparison of the growth of clinical strains and the environment KT2440 on different carbon and nitrogen sources demonstrated that H8234 was the most efficient using as carbon source lipids, hidroxy- and keto- butiric acid, aminoacids as L-glycine. This strain was also able to use efficiently the amino acids serine as and threonine as the only nitrogen source and cysteine as the only sulfur source (Fig 1 and data not shown).

**Fig 2:**
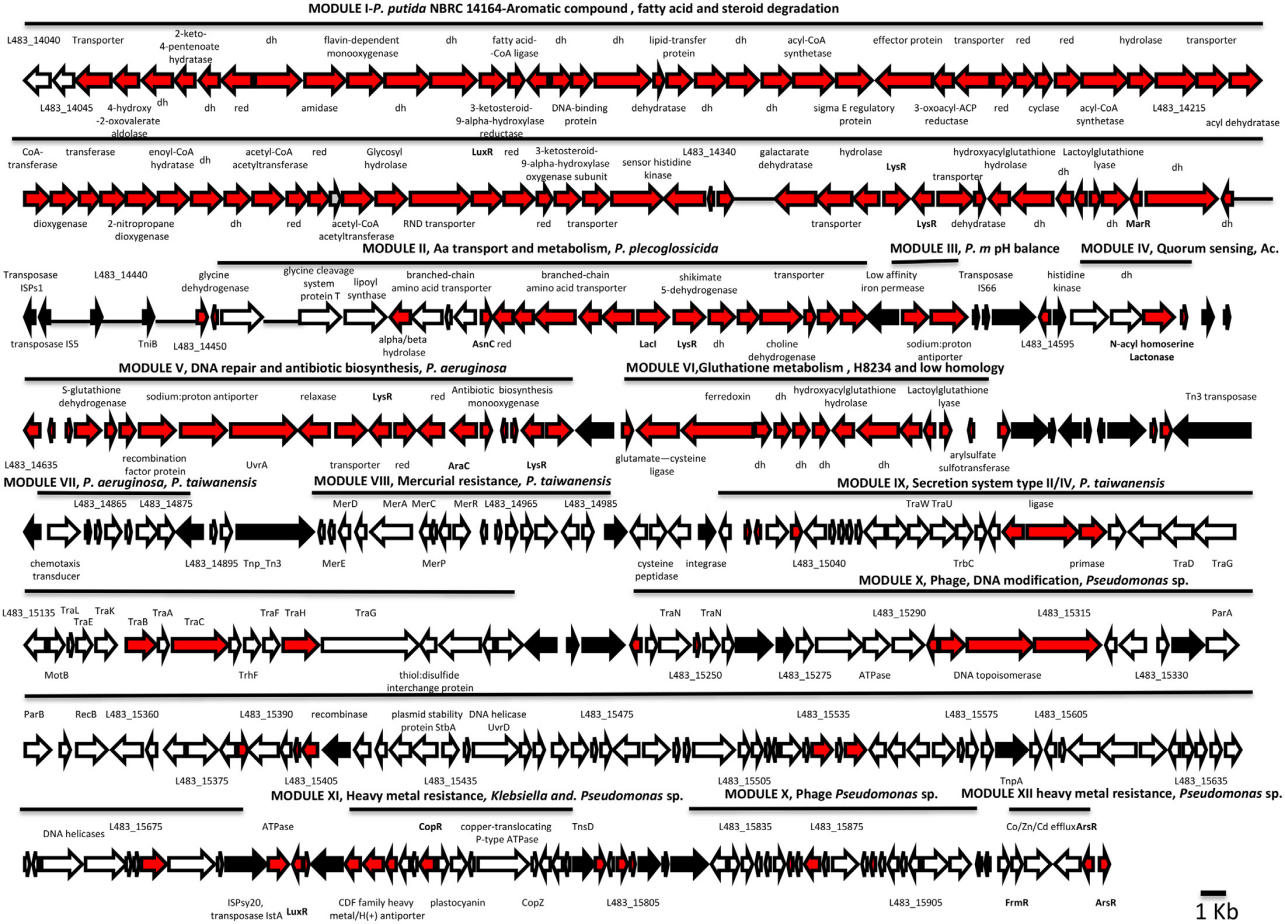
Genetic modules in clusters XVII and XVIII of H8234. Lines represent the length of the modules. Represented in white, genes that have the highest identity with other *Pseudomonas* environmental strains. In red, genes that do not have homology with any other strain or have homology only with pathogens, opportunistic pathogens or clinic isolates of *P*. *putida*. In black, genes involved in transposition events. dh means dehydrogenase, red means oxidoreductase.

This strain also possesses the largest genome of the studied strains (6.9 Mb) (Table 1); approximately 20% of this genome is the result of DNA insertions. H8234 possesses 33 exclusive genetic clusters (S3 Table) (12). The largest segment of the inserted DNA are located in clusters XVII and XVIII, which contain 345 and 138 ORFs, respectively. Both clusters are a mosaic of modules made of transposases and other mobile elements (Fig 2). These modules contain genes that encode proteins found in many different organisms that inhabit multiple niches (edaphic, aquatic and clinical environments, including some pathogens), indicating a horizontal acquisition of these modules (Fig 2). Cluster XVII, the largest, bears 11 modules that contain genes that are important for nutrient uptake and survival under conditions of stress (Fig 2). Of note, the presence of genes involved in steroid/aromatic/lipid (testosterone) transport and degradation is remarkable.

The second largest cluster XVIII (L483_16125-L483_16885) comprises 10 modules that contain genes for iron uptake, resistance to heavy metals, and determinants of tetracycline and gentamycin resistance (S3 Table).

Another feature of the H8234 clusters is the abundance of genes that encode iron uptake systems. Most of the systems are TonB-dependent (clusters VIII, XI, XV, XXII, XXIX and XXXI). These proteins ensure the effective detection of iron (e.g, free Fe^3+^ or molecules such as transferrin, lactoferrin, and hemoglobin) [63].

#### Genes that are specific to HB4184

This strain bears a high number of genes involved in metabolism of amino acids (lysine, threonine, methionine, and cysteine), ABC transporter dipeptide and DNA replication (S2 Table). These features indicate that the major differential evolution strategy used by this strain is related to the metabolism of certain amino acids. HB4184 possesses 34 gene clusters that are not present in any other strains (S3 Table). The most relevant feature is the presence of a type III-like secretion system (ORF3005-15, cluster XXII). The presence of such system has been reported before in strains that belong to the “*P*. *putida* complex” found in blood infections [64]. In pathogenic bacteria, this needle-like structure secretes effector proteins directly from the bacterial cell into the eukaryotic (host) cell, where they exert a number of effects that help the pathogen to survive and to escape an immune response [65]. One possible protein that may be injected (i.e., an effector) could be the protein encoded by ORF3009.

Heat tolerance determinants within this strain appear to be located on a transposon in cluster VI (ORF581-607). These include heat shock proteins Hsp (ORF590-2), HtpX (ORF601) and HtrA (ORF602) that are highly homologous to proteins found in *Pseudomonas pseudoalcaligenes* and *P*. *aeruginosa*. Also located in this transposon are genes involved in survival under stress conditions, including: a thioredoxin involved in maintaining oxidative balance (ORF597); a potassium/proton KefB (ORF598), responsible for pH homeostasis; and the phosphate starvation-inducible PsiE protein (ORF600), which is highly similar to proteins in *Klebsiella pneumoniae* and *P*. *aeruginosa*.

One of the more remarkable characteristics of this strain is the abundance of genes that encode enzymes involved in the detoxification of reactive oxidative species. These proteins are found in cluster XX (ORF2654-2655, peroxiredoxin), XXI (ORF2683, alkyl hydroperoxide reductase and other enzymes involved in preventing free radical damage), XXIX (ORF4572, alkylhydroperoxidase, which offers protection against oxidative and osmotic stress) and XXXIV (ORF5442, alkylhydroperoxidase).

## Discussion

One of the mechanisms used by microbes to adapt to a new niche is the acquisition of the biochemical and biophysical functions required to survive in the new environment [66]. In clinical strains of *P*. *putida* this acquisition appears to have been achieved via horizontal gene transfer mediated by transposons that move a number of genetic traits present in microorganisms that inhabit the niche to be colonized. In the case of *P*. *putida* many of the genes acquired are those related with transposition itself and phage integration. The genetic information associated with these transposons were genes highly homologous in sequence to those in the insect pathogen *P*. *entomophila*, the fish pathogen *P*. *plecoglossicida* and the human opportunistic pathogen *P*. *aeruginosa*. Horizontal gene transfer mediated by transposons has been observed in certain clinical microorganisms, e.g., *P*. *aeruginosa* in order to colonize lungs [24, 67]. In *Enterobacter faecium* the presence of this acquired information could contribute to the transition of an avirulent comensal to a pathogenic form [68]. The most commonly acquired information in the *P*. *putida* clinical strains is related to survival under osmotic and oxidative stress, facing the deleterious effects of biocide molecules, nutritional adaptation and modulation of the human immune response. Below we examined these features in more detail.

### Membrane composition (Phospholipids)

Phospholipids are the major component of bacterial membranes. The phospholipid composition of the membrane is crucial for bacteria to cope with environmental hazards such as extreme pH, high osmolarity, or exposure to surfactant molecules. Moreover, phospholipids play an important role in bacterial infection as they represent both target and barrier for antibiotics and host defense mechanisms such as cationic antimicrobial peptides and enzymes produced by phagocytes or epithelial cells [69]. Genes involved in phospholipid metabolism are overrepresented in the *P*. *putida* clinical isolates compared with environmental strains, the clade II strains stand out in this regard. Specifically, clade II clinical strains (H8234 and HB4184) contain two phospholipid biosynthesis pathways; one of these (L483_11530–90) has a homolog in the entomopathogen *P*. *entomophila*, while the other (L483_17350- L483_17400) is highly conserved in *P*. *putida* strains. As noted, pathogens often have two phospholipid biosynthetic pathways. Although we have no evidence for differential expression, other groups have reported that expression of the additional pathway is only observed during phagocytosis [70].

### Responses against oxidative stress

To successfully infect a host, pathogenic bacteria for animals should be able to overcome many barriers. One primary defense mechanism is the production of reactive oxygen species (ROS) by the host cells, such as, hydroxyl radical (^·^OH), peroxyl radical (ROO^·^), alkoxyl radical (RO^·^), superoxide anion (^·^O^2−^), singlet oxygen (^1^O_2_), hydrogen peroxide (H_2_O_2_) and nitric oxide (NO) [71]. To overcome the detrimental effects of oxidative stress, the invading microorganism must bear cellular defense systems involving the production of ROS detoxification enzymes (such as catalases, peroxiredoxins and superoxide dismutases), as well as bypass the deleterious effect of these ROS in important biomolecules [72–75]. One of the strategies employed by the clinical *P*. *putida* isolates studied here is to increase the number of genes involved in handling oxidative stress compared to the environmental strains. This is especially noticeable for the clade I strain, HB13667 where gene numbers for this functional group are similar to those found in KT2440. KT2440 is able to live in diverse environments and is capable of interacting with various reactive oxygen species-inducing agents existing in numerous niches, for this, the strain has developed a potent equipment against ROS [76].

As mentioned earlier, generation of ROS by the host can cause oxidative modification and damage to important cellular bio-molecules such as lipids, carbohydrates, proteins and DNA via different mechanisms. *De novo* synthesis of the resistance molecules is required for survival under the oxidative conditions [77]. This could be an explanation for the overrepresentation of genes involved in lipid (particularly in clade II, H8234 and the clade I, HB13667), amino acid (especially H8234) and DNA metabolism (HB13667) in *P*. *putida* clinical isolates. The damaged molecules could also be restored. Glutathione plays a crucial role in the restoration of oxidized lipids and proteins [78]. Biosynthetic pathways for this molecule are more frequent in some of the clinical strains H8234 (31 genes) and HB13667 (29 genes) compared with the environmental strains (28 for F1, highest of the environmental strains). Glutathione is a tripeptide composed of glutamate, cysteine, and glycine [77]. Curiously, genes involved in cysteine (HB13667, and the clade II HB4184) and glutamate biosynthetic pathways (clade I, HB3267) are overrepresented in all of the clinical strains. The DNA damage induced by ROS is directly involved in cell death. ROS molecules oxydize nitrogen bases and produce double-strand breaks and single-strand breaks in DNA. The repair mechanisms employ the action of enzymes such as DNA polymerase, helicases (Uvr proteins), and exonoucleases [78]. Genes that encode the mechanisms for DNA repair are overrepresented in clinical isolates of *P*. *putida* (especially in HB13667) compared to environmental strains.

### Responses to the presence of antimicrobial biocides

Antimicrobial agents (antibiotic and heavy metal biocides) have been used traditionally as a treatment or preventative for pathogenic infections. The ability of bacteria to survive in the presence of antibiotics and soluble heavy metals is dependent on the expression of tolerance/resistance genes [79,80]. Although these genes are widely spread in nature, they tend to be more prevalent in clinical isolates [81]. This increased prevalence, specifically of genes involved in heavy metal tolerance and homeostasis, was observed in the set of clinical isolates we characterized when compared with environmental strains. The homology of the genes found in the clinical isolates of this study with the genes found in pathogens and opportunistic pathogens supports their consideration as clinical genes. In fact, genes involved in tolerance to some of these heavy metals (e.g., copper and manganese) have been closely associated with pathogenicity [82, 83]. Of the set of strains studied here, HB13667 is the clinical *P*. *putida* isolate that is the most specialized in the survival in the presence of biocides.

### Maintenance of fitness of the population

One of the mechanisms that bacterial cells use to face stress conditions (oxidative stress, presence of biocides, host defense mechanisms) is the microbial-based production of toxins in order to establish a fit population of cells in a specific environment. Some toxins do so by negatively selecting competing and weaker microorganisms or by acting against host defense mechanisms. For example, toxin-antitoxin (TA) systems are genetic elements composed of a toxin gene and its cognate antitoxin. The antitoxin, expressed by the microbe, serves to neutralize the toxin and enables selective survival. However, under certain circumstances, such as environmental stress, the antitoxin, which is more labile, is degraded more rapidly. As toxin levels rise, the weaker cells undergo cell death, and only the fittest cells survive; this system inhibits the overall growth rate and permits persistence throughout the duration of the stress situation [84]. Among the strains that we analyzed, we found a high sequence diversity in the TA systems, specificity was shown at the niche level (eco-type, clinical and environmental TA systems), and the phenotype level (found only in aromatic degrader or rhizospheric strains). Similar results were found in *Escherichia coli* [85]. Two nonspecific TA systems (RES domain toxin/antitoxin and toxin addition module/toxin) that could be considered general TA systems in *P*. *putida* were also present in most of the studied strains. The MazF/E TA system, that is involved in handling several stress conditions and is also involved in suppression of the immune response [86] is highly diversified in *P*. *putida*; there is a clinical ecotype of this system (i.e., only members of the clinical isolates have homologs), in addition a second variant of this system is also found in the rhizospheric strain, BIRD-1. The MqsR/MqsA TA system, involved in the response to several stress conditions and in biofilm formation [87], appears to constitute an environmental ecotype. The HicA/HicB system which is in involved in survival in the presence of antibiotics [88] shows high diversification in *P*. *putida*, there is an environmental ecotype (present only in environmental strains), a second “bacterial host-related” ecotype present in clinical strains and in W619. In addition, the ChpB/S TA system which is involved in survival under oxidative stress [89] was found in rhizospheric strains and clinical strains of clade I.

### Metabolic and nutritional adaptation

The ability to acquire nutrients during infections is another important attribute in microbial survival in human tissues. For example, amino acids are a valuable source of nitrogen if they can be degraded by the invading organism [90]. It should be noted that histidine is not typically metabolized by *P*. *putida* [91]; however, in the clinical strains analysed here, with the exception of H8234, we found a histidine ABC transport system and a histidine ammonia-lyase that are highly homologous to those found in *P*. *entomophila*. Histidine ammonia-lyase catalyzes the first step of a major histidine degradation pathway in several organisms [92] and may serve to target the abundant histidine-rich glycoproteins found in vertebrate plasma, and which act as a key regulator of the immune response. Not surprisingly, it has been proposed that the degradation of these proteins may provide a source of amino acids for pathogens, while also mediating evasion of the immune response [93]. Other amino acids, such as taurine that is present at high concentrations in most animal tissues, constitute a good source of organic sulfur. This amino acid has an additional role in the protection the tissue from oxidative stress and in the innate immunity [94]. The number of genes involved in organic sulfur metabolism (including taurine utilization genes) is higher in the clinical isolates of *P*. *putida* (especially H8234) than in environmental strains (with exception of GB-1). Another molecule that is abundant in eukaryotes, and forms part of the phosphatidylcholine and sphingomyelin of membrane cells, is choline. Choline is oxidized to glycine betaine and can function as an osmoprotectant, and as a source of carbon, and nitrogen, for human pathogens [95]. Genes involved in choline uptake and betaine biosynthesis are also overrepresented in the clinical isolates of *P*. *putida*. Of note, strain H8234 appears to have the highest metabolic potential to use not only amino acids, but also carbohydrates and organic sulfur metabolites.

### Iron uptake

In order for *P*. *putida* to colonize niches, it must ensure the availability of iron. The source of iron in each niche varies; free Fe^3+^is mostly used by free-living microorganisms because this ion is available in soil [96] and water [97]. However, in animals most of this iron forms part of the hemoproteins [98]. This may be the basis for the acquisition of elaborate high-affinity iron uptake systems by clinical isolates of *P*. *putida*. The main mechanism to capture iron is through the synthesis of siderophores. In *P*. *putida* KT2440 the only siderophore that has been previously identified is pyoverdine [99]. A high level of diversity has been found in the pyoverdine synthesis locus in this genus and species, in fact the diversity is such that it can be used as a good phylogenetic marker [100,101]. We have found the same diversity for this locus, as was described earlier for TA toxin/antitoxin systems; different ecotypes could be described (L. Molina, Z. Udaondo and J. L. Ramos, submitted). Apart from this biodiversity, additional genes involved in pyoverdine biosynthesis or modification have also been found inserted in a transposon in clinical strains (i.e., H8234, L483_16595- L483_16480). These genes are highly homologous to one found in *Pseudomonas alcaligenes* (*P*. *aeruginosa* group). These findings indicate a different evolution in the iron uptake mechanism in clinical *P*. *putida* strains.

### Manipulation of the host environment (Secretion systems)

Pathogens and other components of the human microbiome not only react to their environment, they are also able to manipulate their surroundings and exploit whatever nutrient sources are present by the secretion of proteins and other molecules. Human pathogens have developed a remarkable array of sophisticated nanomachines—secretion systems—to export proteins and DNA into the extracellular environment or into target cells. Two of these secretion systems have evolved in a similar way, the type II (T2S) and type IV (T4S) secretion systems; they are multi-protein complexes spanning the envelope of Gram-negative bacteria and dedicated to the transport of secretion substrates through the bacterial outer membrane in a two-step process [102]. These secretion systems are widely dispersed throughout different species of microorganisms, however, in pathogenic strains it has been shown that there is nearly always more than one T2S or T4S secretion cluster. This appears to be the case for the *P*. *putida* clinical isolates and the endophyte W619. T2S is involved in the degradation of biopolymers, by changing the oxidoreductive state of iron and manganese. In addition, T2S also play an important role during pathogenic bacterial infections by the secretion of virulence factor [103]. One additional cluster encoding a T2S system has been found in clade I strains HB13667 and HB3267 (in HB3267 B479_21275-B479_21345) and is highly homologous to one found in *P*. *plecoglossicida*. T4S transport a diverse array of substrates, from DNA to nucleoprotein complexes and virulence effectors. T4S have been implicated in the conjugation of plasmids carrying antibiotic resistance genes between pathogenic bacteria [104]. An additional T4S was found in clade II strains HB4184 and H8234 (in H8234, L483_30550-L483_30560) and clade I strains (HB3267, B479_07330- B479_07355). The clade I T4S appears to be involved in the secretion of type I fimbriae. Production of fimbriae has been shown to assist human pathogenic microorganisms in attachment to host tissues, and to each other; this attachment allows the subsequent differentiation of the microorganism into a biofilm lifestyle [105]—a structure that increases the resistance to antibiotics and is important for surface colonization and interacting with host factors and the host immune system [106].

A remarkable finding of this study is the identification in the clade II strain HB4184 of other type of nanomachine, a secretion system that can be included in type III (T3S). T3S is frequent found in human and plant pathogenic Gram-negative bacteria, and plays a crucial role in the virulence of these isolates; permitting the export of proteins/effectors into target cells. These effector proteins are able to modulate the immune defenses of the host, producing even cell death [65]. These T3Ss are rarely found in *P*. *putida*, the only mention in scientific reports is a recent study that described the presence of this kind of structure in blood isolates that were assigned to the “*P*. *putida* complex” [64].

In summary, this study describes for the first time, the genetic traits involved in the survival of four clinical isolates of *P*. *putida*. This important genetic information appears to have been acquired by horizontal transfer from pathogenic or opportunistic microorganisms. The newly acquired genes are involved in coping with stress conditions (oxidative stress and presence of biocides traditionally used in the hospital environment), the uptake of new nutrients available in the host tissues (C, N, S and iron sources), and modification of the host environment (abolishing host defense responses). These four clinical isolates present different characteristics: HB13667 appears to be more equipped at coping with oxidative stress and biocides, HB3267 is a highly antibiotic resistant strain [9], HB4184 possesses a likely type III secretion system and H8234 appears to be adapted to survive in highly differential environments, having a high metabolic potential. The complexity found in this last strain is due to the large amount of acquired DNA, where approximately 20% has been received by horizontal gene transfer from other microorganisms living in environmental and clinical environments.

## Supporting Information

S1 Fig Phylogenetic relationships (determined using the GyrB, Edd, RecA, TrpF and RpoD protein sequence) between the different *Pseudomonas* strains. Arrows indicate clinical strains, continuous arrows clade I strains, discontinuous arrows clade II strains; within the black square are strains in the *P*. *putida* group.
(PPTX)

S2 Fig Phylogenetic relationships between the different *Pseudomonas* strains (determined using complete genomes). (PPTX)

S1 Table TA systems and pyocins found in *P*. *putida* strains. (DOCX)

S2 Table Functional categories predominant in each clinical *P*. *putida* isolate. (DOCX)

S3 Table Gene clusters that are specific to each *P*. *putida* clinical strain. (DOCX)
