# Supplementary figures and images for: Specific Gene Loci of Clinical Pseudomonas putida Isolates
Source: PLoS One. 2016 Jan 28;11(1):e0147478. doi: 10.1371/journal.pone.0147478 (PMC4731212; doi:10.1371/journal.pone.0147478)

## Slide 1
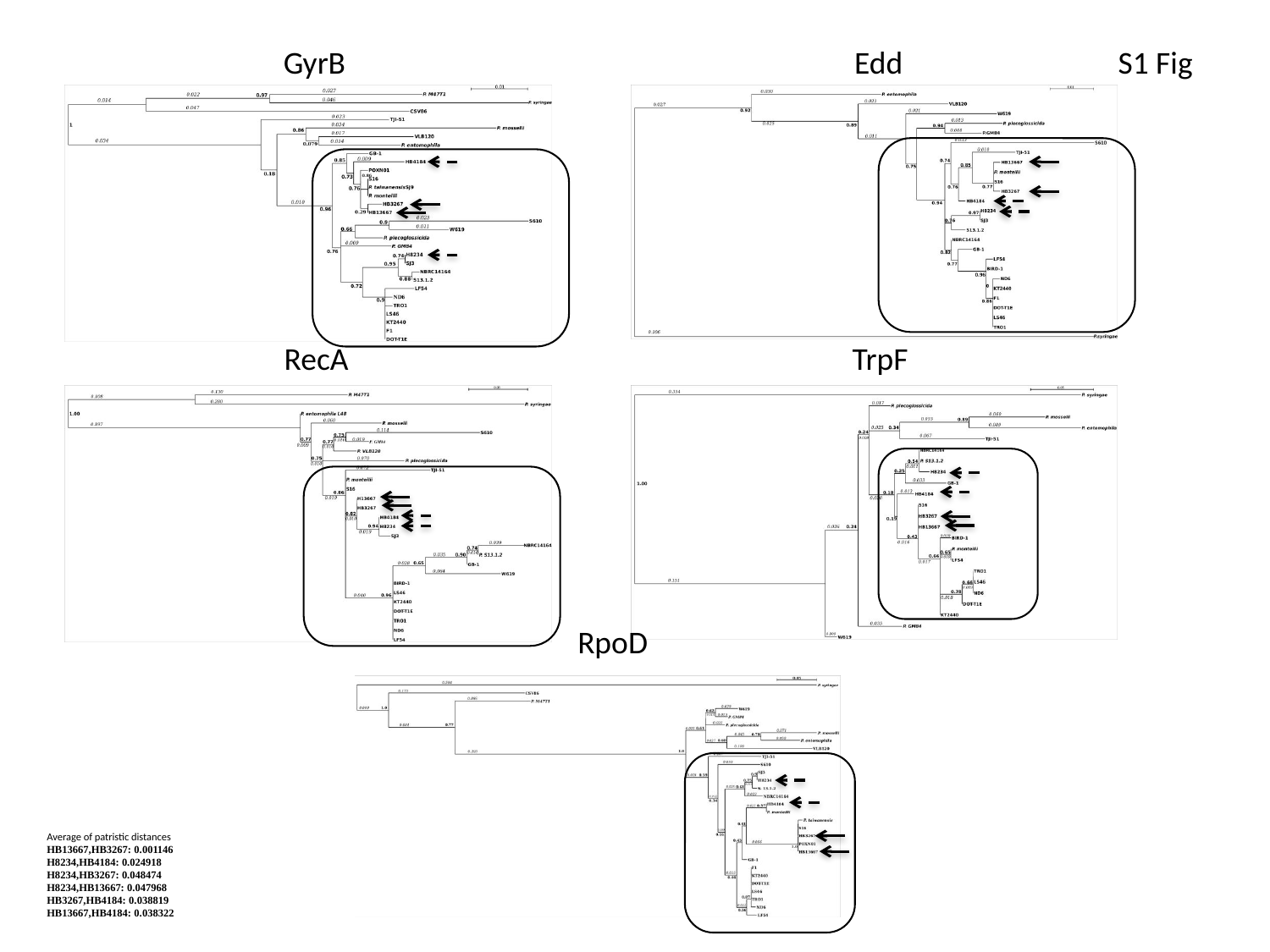

GyrB
Edd
RecA
TrpF
S1 Fig
RpoD
Average of patristic distances
HB13667,HB3267: 0.001146
H8234,HB4184: 0.024918
H8234,HB3267: 0.048474
H8234,HB13667: 0.047968
HB3267,HB4184: 0.038819
HB13667,HB4184: 0.038322

Supplement: S1 Fig — Arrows indicate clinical strains, continuous arrows clade I strains, discontinuous arrows clade II strains; within the black square are strains in the P. putida group. (PPTX) [file pone.0147478.s001.pptx]

## Slide 1
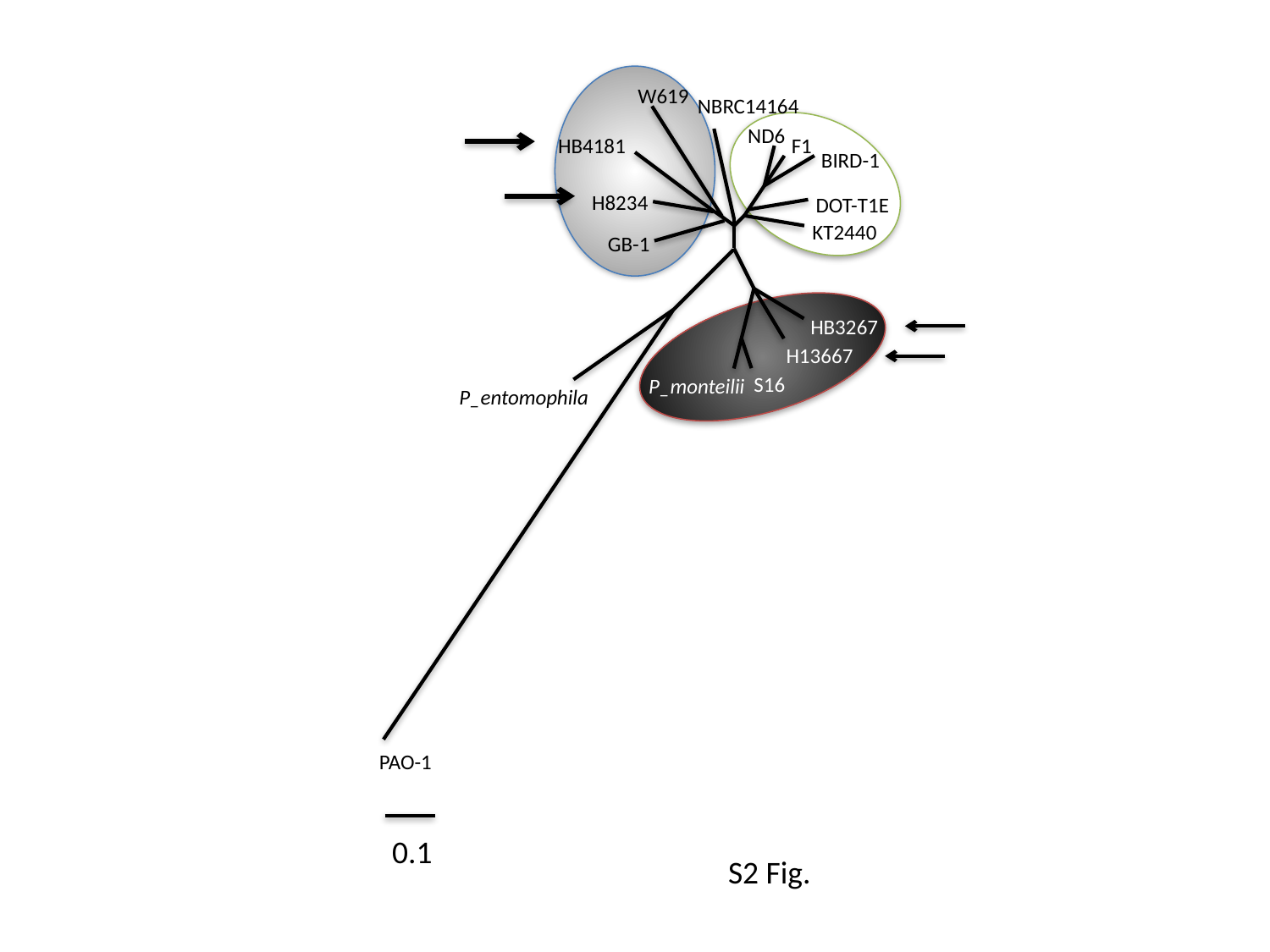

W619
NBRC14164
ND6
HB4181
F1
BIRD-1
H8234
DOT-T1E
KT2440
GB-1
HB3267
H13667
S16
P_monteilii
P_entomophila
PAO-1
0.1
S2 Fig.

Supplement: S2 Fig — (PPTX) [file pone.0147478.s002.pptx]
