# Supplementary material for: Specific Gene Loci of Clinical Pseudomonas putida Isolates
Source: PLoS One. 2016 Jan 28;11(1):e0147478. doi: 10.1371/journal.pone.0147478 (PMC4731212; doi:10.1371/journal.pone.0147478)
Supplement: S1 Table — (DOCX) [file pone.0147478.s003.docx]

S1 Table . TA systems and pyocins found in *P. putida* strains

|  | **KT2440** | **BIRD-1** | **S16** | **W619** | **F1** | **DOT-T1E** | **GB-1** | **HB3267** | **H8234** | **HB13667** | **HB4184** | **Closest relative in exclusive genes** |
| --- | --- | --- | --- | --- | --- | --- | --- | --- | --- | --- | --- | --- |
| **TA systems** |  |  |  |  |  |  |  |  |  |  |  |  |
| Antitoxin-MazE/ChpS | PP_0770 | PPUBIRD1_0816 | PPS_0826 | PputW619_4419 |  |  |  | B479_04340 |  | ORF1364 |  |  |
| Toxin ChpB | PP_0771 | PPUBIRD1_0817 | PPS_0827 | PputW619_4418 |  |  |  | B479_04345 |  | ORF1363 |  |  |
| Toxin_YhaV |  |  |  |  |  | T1E_1581 |  |  |  |  | ORF2557 |  |
| Antitoxin MazE family |  |  |  |  |  | T1E_1582 |  |  |  |  | ORF2556 |  |
| Antitoxin-MazE, AbrB |  | PPUBIRD1_2909 |  |  |  |  |  |  | L483_11385 |  | ORF3002 |  |
| Toxin YhaV |  | PPUBIRD1_2910 |  |  |  |  |  |  |  |  |  | RPPX_24090 |
| AntitoxinVapC |  |  |  | PputW619_4624 |  |  |  |  |  |  |  | *Pseudomonas plecoglossicida* NB2011 |
| Toxin VagC/MazE |  |  |  | PputW619_4623 |  |  |  |  |  |  |  | *Pseudomonas plecoglossicida* NB2011 |
| Antitoxin MazE |  |  |  |  |  |  |  | B479_25740 | L483_32130 |  | ORF2099 |  |
| Toxin |  |  |  |  |  |  |  | B479_25735 |  |  |  |  |
|  |  |  |  |  |  |  |  |  |  |  |  |  |
| Antitoxin RelB |  |  |  |  | Pput_1207 | T1E_4039 |  |  |  |  | ORF0289 |  |
| Toxin RelE |  |  |  |  | Pput_1208 |  |  |  |  |  |  |  |
| Antitoxin RelB |  |  |  |  |  |  | PputGB1_4037 |  | L483_24745 |  |  |  |
| Toxin RelE |  |  |  |  |  |  | PputGB1_4036 |  | L483_24740 |  |  |  |
| Toxin RelE |  |  |  |  |  | T1E_4038 |  |  |  |  | ORF0290 |  |
| Toxin RelE |  |  |  | PputW619_4958 |  |  |  | - |  |  |  | *Pseudomonas* sp. GM84 |
| Toxin RelE |  |  | PPS_2078 |  |  |  |  | B479_10835 |  | ORF3162 | ORF3887 |  |
| Antitoxin Phd/YeFM |  |  | PPS_2077 |  |  |  |  | B479_10830 |  | ORF3163 |  |  |
| Antitoxin |  |  |  |  |  |  |  | B479_01855 |  | ORF5143 |  |  |
| Toxin RelE |  |  |  |  |  |  |  |  |  |  |  | *Pseudomonas monteilii* |
| Antitoxin addition module | PP_1241 | PPUBIRD1_1279 |  |  | Pput_1271 |  |  |  | L483_25210 |  |  |  |
| Toxin |  | PPUBIRD1_1280 | PPS_3549 | PputW619_3102 | Pput_1272 | T1E_5044 | PputGB1_3688 | B479_11135 | L483_25205 |  | ORF4342 |  |
| Toxin HicA-1 | PP_1480 |  | PPS_1132 |  |  |  |  |  |  |  |  |  |
| Antitoxin HicB-1 | PP_1479 | PPUBIRD1_4078 | PPS_1133 | PputW619_4138 | Pput_4242 | T1E_2857 |  |  |  |  |  |  |
| Antitoxin HicB |  |  |  | PputW619_3853 |  |  |  | B479_19385 |  | ORF5512 |  |  |
| Toxin HicA |  |  |  | PputW619_3854 |  |  |  | B479_19390 |  | ORF5511 |  |  |
| Toxin hicA-2 | PP_3900 |  |  |  |  |  |  |  |  |  |  | *Pseudomonas syringae* |
| Antitoxin hicB-2 | PP_3899 |  |  |  |  |  |  |  |  |  |  | *Pseudomonas syringae* |
| Antitoxin HigA protein |  |  |  |  |  |  | PputGB1_3643 |  |  | - |  | *Pseudomonas* sp. C5pp |
| Toxin HigB |  |  |  |  |  |  | PputGB1_3644 |  |  | - |  | *Pseudomonas* sp. 10-1B |
| Antitoxin HipB | PP_1585 |  |  | PputW619_4087 | Pput_4192 |  |  |  |  |  |  |  |
| killer protein | PP_1586 |  |  |  | Pput_4191 |  |  |  |  |  |  |  |
| antitoxin | PP_2433 | PPUBIRD1_3249 | PPS_3284 |  | Pput_3262 | T1E_5246 | PputGB1_2078 | B479_16335 | L483_19960 | ORF0716 |  |  |
| RES domain toxin | PP_2434 | PPUBIRD1_3248 | PPS_3283 |  | Pput_3261 | T1E_5245 | PputGB1_2079 | B479_16330 | L483_19955 | ORF0715 |  |  |
| Antitoxin Phd_YefM | PP_2498 |  |  |  |  |  |  |  |  |  |  | *Pseudomonas aeruginosa* DHS01 |
| Toxin | PP_2499 |  |  |  |  |  |  |  |  |  |  | *Pseudomonas aeruginosa* DHS01 |
| Antitoxin Phd_YefM | PP_2940 |  |  |  |  |  |  |  |  |  |  | *Pseudomonas monteilii* |
| toxin YoeB | PP_2939 |  |  |  |  |  |  |  |  |  |  | *Pseudomonas monteilii* |
| Antitoxin MqsA | PP_4204 | PPUBIRD1_1646 |  | PputW619_3528 | Pput_1649 | T1E_0446 | PputGB1_3776 |  |  |  |  |  |
| mRNA interferase MqsR | PP_4205 | PPUBIRD1_1645 |  | PputW619_3529 | Pput_1648 | T1E_0447 | PputGB1_3777 |  |  |  |  |  |
| **Total** | **9** | **6** | **5** | **6** | **6** | **5** | **5** | **6** | **5** | **5** | **6** |  |
| **Pyocin** |  |  |  |  |  |  |  |  |  |  |  |  |
| pyocin S-type Killer | PP_1306 |  |  |  |  |  |  | B479_22065 |  |  |  |  |
| Pyocin S-type immunity | PP_1305 |  |  |  | Pput_4420 |  |  |  |  |  |  |  |
| Pyocin_S |  | PPUBIRD1_4954 |  |  |  |  |  |  |  |  |  |  |
| Colicin/pyocin immunity |  | PPUBIRD1_4955 |  |  |  |  |  |  |  |  |  |  |
| S-type pyocin |  |  |  | PputW619_0912 |  |  |  | B479_04340 |  |  |  | *Pseudomona*s sp. URMO17WK12:I8 |
| hypothetical |  |  |  | PputW619_0913 |  |  |  |  |  |  |  | *Pseudomonas plecoglossicida* NB2011 |
| S-type Pyocin |  |  |  |  |  |  | PputGB1_5377 |  |  |  |  | *Pseudomonas* sp. 10-1B |
| S-type Pyocin |  |  |  |  |  |  | PputGB1_5378 |  |  |  |  | *Pseudomona*s sp. 10-1B |
| S-type Pyocin |  |  |  |  |  |  | PputGB1_2036 |  |  |  |  | *Pseudomonas* sp. GM74 |
| S-type pyocin |  |  |  |  |  |  | PputGB1_4543 |  | L483_27135 |  |  |  |
| pyocin immunity protein |  |  |  |  |  |  | PputGB1_4544 |  | L483_27140 |  |  |  |
| S-type Pyocin |  |  | PPS_5012 |  |  |  |  |  |  |  |  | *Pseudomonas monteilii* SB3078 |
| colicin immunity protein |  |  | PPS_5013 |  |  |  |  |  |  |  |  | *Pseudomonas monteilii* SB3078 |
| S-type Pyocin |  |  |  |  |  |  |  | B479_01965 |  | ORF5165 |  | *-* |
| pyocin immunity protein |  |  |  |  |  |  |  | B479_01960 |  | ORF5164 |  | *Pseudomonas plecoglossicida* |
| pyocin immunity protein | - |  |  |  |  |  |  | B479_22070 |  | ORF4026 |  | *Enterobacter asburiae* |
| S-type Pyocin |  |  |  |  |  |  |  | B479_22065 |  | ORF4027 |  | PP_1306 |
| **Total** | **1** | **1** | **1** | **1** | **1** | **0** |  | **3** | **1** | **2** | **0** |  |

Represented in green the “rhizospheric”/clade I ecotype, in red clinical ecotype TA systems, in blue degrader ecotype, in yellow generic TA systems, in brown environmental ecotypes, in light red clade I ecotype. In grey strain specific TA systems and pyocines.
