# Supplementary material for: Specific Gene Loci of Clinical Pseudomonas putida Isolates
Source: PLoS One. 2016 Jan 28;11(1):e0147478. doi: 10.1371/journal.pone.0147478 (PMC4731212; doi:10.1371/journal.pone.0147478)
Supplement: S2 Table — (DOCX) [file pone.0147478.s004.docx]

**S2 Table. Functional categories predominant in each clinical *P. putida* isolate.**

Numbers indicate the genes involved in a determined functional category. In grey, categories predominant in each clinical isolate.
