# Supplementary material for: Specific Gene Loci of Clinical Pseudomonas putida Isolates
Source: PLoS One. 2016 Jan 28;11(1):e0147478. doi: 10.1371/journal.pone.0147478 (PMC4731212; doi:10.1371/journal.pone.0147478)
Supplement: S3 Table — (DOCX) [file pone.0147478.s005.docx]

S3 Table. Gene clusters that are specific to each *P. putida* clinical strain

| Clusters | Coordinates | Inserted | Function |
| --- | --- | --- | --- |
|  | **HB3267** |  |  |
| I | B479_00025-B479_00140 | Tn7-like transposon | Oxidoreduction processes, amino acid metabolism and transport, |
| II | B479_03665-B479_03675 |  | DNA modification |
| III | B479_03710-B479_03725 |  | Unknown |
| IV | B479_06565 |  | Unknown |
| V | B479_06860-B479_06865 |  | Lipid metabolism |
| VI | B479_06880-B479_06990 |  | Antibiotic resistance, lipid metabolism, sugar metabolism, amino acid metabolism |
| VII | B479_08470-B479_08760 | integrases | Phage |
| VIII | B479_08775 |  | Unknown |
| IX | B479_08800-B479_08830 |  | Unknown |
| X | B479_10075-B479_10515-B479_10625 | integrases | Defense mechanisms; Stress resistance, DNA repair |
| XI | B479_12425 |  | Unknown |
| XII | B479_12970-B479_13085 |  | Transport, lipid metabolism, defense mechanisms,  Stress response, aromatic degradation, |
| XIII | B479_13735-B479_13750 |  | DNA repair |
| XIV | B479_13955-B479_13985 | integrase | Unknown |
| XV | B479_16720-B479_16915 |  | Phage |
| XVI | B479_18740-B479_18800 |  | mobility, fatty acid metabolism |
| XVII | B479_19770-B479_20185 | integrases | Phage |
| XVIII | B479_21335-B479_21340 |  | Unknown |
| XIX | B479_23080-B479_23180 | transposase | DNA repair and metabolism, virulence, |
| XX | B479_25745-B479_25935 | transposaseTn7_Tnp_TnsA_N; TnsA endonuclease N terminal | DNA repair, transport and aromatic degradation |
| XXI | B479_26570-B479_26585 |  | Transport, oxidoreductation. |
|  |  |  |  |
|  | **H8234** |  |  |
| I | L483_01110-L483_01365 | Integrases | Phage |
| II | L483_02500-L483_02545 | Integrases | Stress response, transport |
| III | L483_03340-L483_03355 |  | oxidative stress |
| IV | L483_03390-L483_03405 |  | Stress response |
| V | L483_06415-L483_06450 |  | Lipid metabolis |
| VI | L483_08095-L483_08625 | recombinases | Phage |
| VII | L483_09025-L483_09145 | transposases | Carbon metabolism, stress response, amino acid metabolism,  sulfur metabolism, transport |
| VIII | L483_10575-L483_10610 |  | Iron uptake, defense |
| IX | L483_11080-L483_11110 |  | Stress response, transport |
| X | L483_11370-L483_11480 |  | Osmotic stress, sugar and amino acid metabolism/ transport |
| XI | L483_11595-L483_11605 |  | Iron uptake |
| XII | L483_12010- L483_12030 | recombinase, transposase | Transport of branched chain amino acids |
| XIII | L483_12250-L483_12505 | integrases, transposase | phage, DNA repair |
| XIV | L483_12910-L483_13355 | integrases | Phage |
| XV | L483_13500-L483_13510 |  | Iron uptake |
| XVI | L483_13625-L483_13700 |  | Sulfur metabolism, aromatic degradation, defense, amino acid metabolism |
| XVII | L483_14040-L483_15950 | transposases | Carbon metabolism, lipid metabolism, aromatic degradation,  transport, virulence, oxidative stress, iron, aminoacid metabolism and transport  antibiotic biosynthesis, biocide resistance, DNA metabolism |
| XVIII | L483_16125-L483_16885 | transposases, integrases, | DNA metabolism, virulence, biocide resistance, transport, aromatic amino acid metabolism and transport, iron uptake, oxidative stress, lipid metabolism |
| XIX | L483_17490-L483_17510 |  | Aromatic compounddegradation |
| XX | L483_19570-L483_19585 |  | Aminoacid metabolism |
| XXI | L483_19740-L483_19765 | integrase | Carbon metabolism |
| XXII | PP4_19570-L483_20065 |  | Iron uptake, amino acid amino acid metabolis |
| XXIII | L483_20240-L483_20275 |  | DNA repair, phage |
| XXIV | L483_20380-L483_20715 | integrase | Phage |
| XXV | L483_20945-L483_21140 | integrases | Phage |
| XXVI | L483_21275-L483_21320 |  | Transport , aromatic compound degradation |
| XXVII | L483_21845-L483_21895 | integrase and transposase | Transport |
| XXVIII | L483_21975-L483_22040 |  | Lipid metabolism, sulfur metabolism,  degradation of aromatic compounds |
| XXIX | L483_22165-L483_22175 |  | Iron uptake |
| XXX | L483_22720-L483_22820 |  | DNA metabolism, degradation and transport of aromatic compounds, amino acid transport, |
| XXXI | L483_25160-L483_25170 |  | Iron uptake |
| XXXII | L483_25845-L483_25865 | integrase | Phage |
| XXXIII | L483_28275-L483_28570 | integrases | phage, biocide resistance |
|  |  |  |  |
|  | **HB13667** |  |  |
| I | 170-257 | transposases | Amino acid metabolism, DNA metabolism, iron uptake, virulence, oxidative stress |
| II | 418-476 | transposases | oxidative stress, mobility, (glutathione metabolism)-  biocide resistance, iron uptake, amino acid metabolism and transport |
| III | 596-622 | transposases | DNA repair and modification, biocide resistance, lipid metabolism,  phosphate transport, virulence, oxidative strees, sulfur metabolism |
| IV | 785-811 | recombinases | phage , DNA metabolism, |
| V | 1106-1197 | transposases, recombinases | Metabolism and transport amino acids, biocides production,  DNA metabolism, |
| VI | 1286-1321 | integrases | Biocide resistance |
| VII | 1456-1520 |  | Phage |
| VIII | 1531-1581 | integrases | Phage |
| IX | 1609-1668 | integrases | Virulence, DNA metabolism |
| X | 1772-1786 | integrases | Carbon and nitrogen metabolism, oxidative stress |
| XI | 2257-2294 |  | Phage |
| XII | 3030-3061 | integrases and transposases | DNA repair, aromatic compound degradation, transport |
| XIII | 3202-3234 |  | Phage |
| XIV | 3301-3370 |  | Aromatic compound metabolism, oxidative stress |
| XV | 3638-3647 | integrases-transposase | Phage |
| XVI | 4527-4548 | transposases | Lipid metabolism |
| XVII | 4897-4909 | transposases | Stress response |
| XVIII | 5003-5025 | integrases | Phage |
| XIX | 5681-5699 | transposase-integrase | Phage |
| XXI | 5790-5817 |  | Phage |
|  |  |  |  |
|  | **HB4184** |  |  |
| I | 8-29- |  | Phage |
| II | 96-134 |  | Phage |
| III | 193-196 |  | Iron uptake, virulence |
| IV | 239-268 |  | amino acid metabolism and transport , aromatic compound degradation, DNA metabolism, |
| V | 339-389 | integrases | Virulence, DNA metabolism, stress, sulfur metabolism |
| VI | 581-610 | transposases | Stress response |
| VII | 628-636 | integrases | Unknown |
| VIII | 677-709 | integrases | Virulence, iron uptake |
| IX | 934-951 | transposases-integrase | Unknown |
| X | 1036-1044 | integrase | Unknown |
| XI | 1512-1515 | relaxase-recombinase | Unknown |
| XIII | 1572-1607 | transposase | DNA metabolism, biocide resistance, oxidative stress, amino acid metabolism and transport |
| XIV | 1710-1713 |  | Metabolism of amino acids, oxidative stress |
| XV | 1919-1922 |  | Transport |
| XVI | 2018-2061 | integrase | Phage |
| XVII | 2096-2187 | integrase-recombinase | Phage |
| XVIII | 2422-2424 |  | DNA metabolism |
| XIX | 2517-2524 |  | Phage |
| XX | 2654-2655 |  | Oxidative stress |
| XXI | 2680-2687 |  | Oxidative stress, DNA metabolism |
| XXII | 3002-3015 |  | Virulence |
| XXIII | 3555-3560 |  | Virulence |
| XXIV | 3592-3614 |  | Lipid metabolism |
| XXV | 3845-3848 |  | Virulence |
| XXVI | 4081-4093 |  | Oxidative stress, virulence |
| XXVII | 4343-4387 | integrases | Phage |
| XXVIII | 4402-4413 |  | DNA metabolism |
| XXIX | 4567-4596 | transposase | Oxidative stress, hormone transport, transport, DNA metabolism, amino acid metabolism, degradation of aromatic compounds, virulence |
| XXX | 4610-4617 |  | Virulence |
| XXXI | 4827-4829 | transposases | Viruelnce |
| XXXII | 5141-5185 | recombinases | DNA metabolism |
| XXXIII | 5413-5414 |  | Iron uptake |
| XXXIV | 5423-5462 | integrases transposases | Virulence, resistance to biocides, amino acid metabolism, |
